# Waterfalls drive parallel evolution in a freshwater goby

**DOI:** 10.1002/ece3.295

**Published:** 2012-07-01

**Authors:** Yuichi Kano, Shin Nishida, Jun Nakajima

**Affiliations:** 1 Graduate School of Engineering, Kyushu UniversityFukuoka, Japan; 2 Graduate School of Social and Cultural Studies, Kyushu UniversityFukuoka, Japan; 3 Fukuoka Institute of Health and Environmental SciencesDazaifu, Fukuoka, Japan

**Keywords:** Allopatric speciation, Iriomote Island, land erosion, landlocked fish, *Rhinogobius* sp. YB, *Rhinogobius brunneus* (*Rhinogobius* sp. DA)

## Abstract

Waterfalls may affect fish distribution and genetic structure within drainage networks even to the extent of leading evolutionary events. Here, parallel evolution was studied by focusing on waterfall and the landlocked freshwater goby *Rhinogobius* sp. YB (YB), which evolved from amphidromous *R. brunneus* (BR). The fish fauna was surveyed at 30 sites in 11 rivers on Iriomote Island, Japan, the geography of which was characterized by terraces/tablelands with many waterfalls. We found that all YB individuals were distributed only above waterfalls (height 6.8–58.7 m), whereas BR, and other fishes, were mostly distributed below waterfalls. Mitochondrial DNA analysis showed that every YB local population above the waterfall was independently evolved from BR. In contrast, cluster analysis of nine morphological characters, such as fin color and body pattern, showed that the morphology of YB individuals held a similarity beyond the genetic divergence, suggesting parallel evolution has occurred relating to their morphology. Genetic distance between each YB local population and BR was significantly correlated with waterfall height (*r*^2^ = 0.94), suggesting that the waterfalls have been heightened due to the constant geological erosion and that their height represents the isolation period of YB local populations from BR (ca. 11,000–88,000 years). Each local population of BR was once landlocked in upstream by waterfall formation, consequently evolving to YB in each site. Although the morphology of YB had a high degree of similarity among local populations, finer scale analysis showed that the morphology of YB was significantly correlated with the genetic distance from BR. Consequently, there could be simultaneous multiple phases of allopatric/parallel evolution of the goby due to variations in waterfall height on this small island.

## Introduction

Geographic isolation is one of the causes of speciation. Biological populations of identical species may become isolated by geographic change, such as mountain building, or social change, such as emigration, leading to changes in selectivity, mutation, and/or genetic drift; this eventually leads to the evolution of distinctly different characteristics (Dobzhansky 1951; Mayr 1963; Coyne and Orr 2004).

Of the various types of evolution/speciation, parallel evolution refers to the independent development of a similar character starting from a same ancestral condition (Zhang and Kumar 1997). In freshwater fishes, parallel evolution has often been discussed in the three-spined sticklebacks (genus *Gasterosteus*), where a stream-resident ecotype (small size, low armor) has repeatedly evolved from an anadromous ecotype (large size, high armor) (Hagen and MacPhail 1970; Rundle et al. 2000; Taylor and McPhail 2000; Reusch et al. 2001; McKinnon et al. 2004). The reproductive isolation of the stream-resident and anadromous ecotypes occurred as a result of size-assortative mating, that is, large individuals tended to prefer large mates, whereas small individuals tended to prefer small mates (McKinnon et al. 2004).

Waterfalls have occasionally been studied in ecology as physical barriers to fish migration and factors that may affect fish distribution and genetic structure in drainage networks. Habitats above waterfalls are low predation sites for guppies, whereas those below waterfalls are high predation sites; thus, the colorfulness of males is associated with a trade-off between predation risk and sexual appeal (e.g., Endler 1980; Endler and Houde 1995; Magurran 2005; Crispo et al. 2006; Labonne and Hendry 2010). The genetic diversity of salmonid populations above waterfalls is low because of the unidirectional gene flow and isolation (e.g., Carlsson and Nilsson 2001; Castric et al. 2001; Costello et al. 2003; Guy et al. 2008). Habitats above waterfalls are refugia lacking predatory fishes, and this enhances survival following the upstream migration of Hawaiian stream goby (*Sicyopterus stimpsoni*) (Blob et al. 2010) and diadromous shrimps (*Atya lanipes* and *Xiphocaris elongate*) (Covich et al. 2009).

*Rhinogobius brunneus* (equal to *Rhinogobius* sp. DA [Oijen et al. 2011]) is an amphidromous goby, which is widely distributed among rivers/streams in Japan except northern Hokkaido Island (Mizuno 2005). The fish spawns small eggs (long diameter of ellipsoid yolk sac: 2.4–2.5 mm) from May to July, and the fries go to sea where they grow as larvae before migrating back into the rivers (Nishijima 1968; Mizuno 2005). *Rhinogobius* sp. YB (yellow belly type), a freshwater goby, is distributed in the mountain streams of the Ryukyu Archipelago, Japan (Iwata 2005). The fish spawn middle size eggs (long diameter of ellipsoid yolk sac: ca. 5.1 mm) in early summer. The larva of this fish is extremely intolerant of salinity (Hirashima and Tachihara 2000); therefore, the species is unlikely to have spread to neighboring rivers through the sea (Ohara et al. 2008). Nishida (1994) proposed an hypothesis related to the evolution of *R*. sp. YB, whereby amphidromous *R. brunneus* individuals reaching the upper reaches of river systems, became isolated and evolved independently, eventually producing a landlocked species; however, no data/evidence were provided to support this. In another study, microsatellite and mitochondrial DNA (mtDNA) analyses of six *R*. sp. YB populations from four islands in the Ryukyu Archipelago partially supported Nishida's hypothesis, although the results and conclusion were still unclear (Ohara et al. 2008).

Iriomote Island is southwest of the Ryukyu Archipelago. The island is mountainous and has numerous rivers, which are inhabited by various fishes, including *R*. sp. YB and *R. brunneus*. Waterfalls are part of the characteristic geography of the island. In this study, fluvial fishes were broadly surveyed in the rivers of the island, with particular attention to waterfalls as a factor determining fish distribution. We further focused on the genetics and morphology of *R*. sp. YB and *R. brunneus*. The results indicated that waterfalls have an ecological role in the distribution of fishes of the island by preventing the migration of amphidromous fishes and in the independent evolution of *R*. sp. YB local populations that were landlocked by historical waterfall formation events. We suggest that this is a case of parallel evolution. In addition, the evolution of *R*. sp. YB was associated with waterfall heights that represented the land erosion and isolation period of *R*. sp. YB local populations.

## Materials and Methods

### Study sites

**Figure 1:**
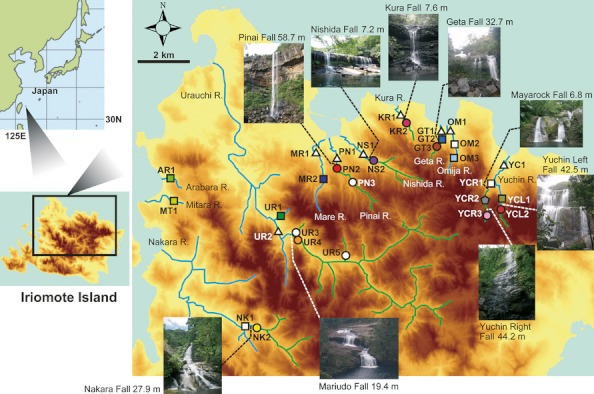
Map and photographic images of waterfalls on Iriomote Island, Japan. Rivers are represented as blue (below waterfall) and green (above waterfall) lines. The study sites are indicated with circles (*Rhinogobius* sp. YB distribution), squares (R. brunneus distribution), triangles (neither *R*. sp. YB nor *R. brunneus* distribution), and pentagon (both *R*. sp. YB and *R*. *brunneus* distribution). White symbols indicate sites that were not sampled for phylogenetic or morphological analyses.

This study was conducted on Iriomote Island, Japan, which is located 200 km east of Taiwan (Fig. 1; 24°21'30”N, 123°50'30”E). The island has a surface area of 289 km^2^, 90% of which is covered by subtropical rainforest, and has an average elevation of 149.0 m (range, 0.10–464.1 m; standard deviation, 113.9 m; based on 10-m mesh digital elevation model data for the island; Geospatial Information Authority Japan 2012).

A total of 30 study sites (each 50 m long) were established along 11 rivers on the northern part of the island (Fig. 1). The study sites were established to test the effect of the waterfalls (Table 1) upon fish distribution. Thus the sites were basically situated neighboring (just above and below) the waterfalls. Several sites were also established where no such waterfalls were present. Site YCR2 was the only site situated between two waterfalls.

**Table 1:** Property of the surveyed waterfalls in the Iriomote Island, Japan

Name	River system	Number of steps	Total vertical height (m)	Waterfall age estimated (year)
Nakara Fall	Nakara R.	2	27.9	41,858
Mariudo Fall	Urauchi R.	3	19.4	29,106
Pinai Fall	Pinai R.	1	58.7	88,067
Nishida Fall	Nishida R.	2	7.2	10,802
Kura Fall	Kura R.	2	7.6	11,402
Geta Fall	Geta R.	3	32.7	49,060
Mayarock Fall	Yuchin R. (right stem)	2	6.8	N/A^1^
Yuchin Right Fall	Yuchin R. (right stem)	3	44.2	66,313
Yuchin Left Fall	Yuchin R. (left stem)	5	42.5	63,763

1 Not available for Mayarock Fall.

### River environment, waterfalls, and fish fauna

In this study, we aimed to test assumptions that the waterfalls prevent the upstream migration of amphidromous/brackish fishes and the distribution of *Rhinogobius* sp. YB was restricted only above waterfalls. Thus, a niche analysis was conducted with a relationship between several environmental factors including presence/absence of waterfalls and the distribution of the fishes.

The assessment of the river environment was conducted in 5–15 June 2011. The weather was generally fine and the river condition was stable during the period. Ten factors were used to evaluate the environment at the site (Table 2). Presence/absence of waterfall (> 3 m height) below the sites was confirmed at the field. Watershed area, elevation, and distance from sea were obtained using ArcGIS 9.3 (ESRI Japan). Water depth, water velocity, and riverbed pebble size were measured every three paces while walking a zig-zag line in the river (Bevenger and King 1995). Water depth and pebble size were measured by a 1-mm scaled staff pole. Water velocity was measured by a portable velocity indicator (VR-301; KENEK). River width was measured at 10-m intervals using a laser range-finder (TruPulse 200; Laser Technology). Photographs were taken in mid-stream at 10-m intervals using a digital camera with a fisheye lens, and the canopy cover was determined using image processing software. The average of each factor at each site was used as the representative value of the site. The slope angle at a 50-m scale was measured using a laser range-finder.

**Table 2:** Ten environmental factors and their coefficient (SE) from GLM/GLMM model selection by AIC for respective species/taxon and species richness

Independent variable	*Rhinogobius* sp. YB	*Rhinogobius brunneus*	*Rhinogobius* sp. CB	*Rhinogobius* sp. DL	Sicydiaphiinae spp.	*Kuhlia* spp.	Species richness
Waterfall (dummy variable)	6.8 (2.1)^**^	–2.3 (1.1)^*^	–17.86 (9.7)		–5.73 (2.3)^*^	–7.0 (3.9)	–1.291 (0.20)^***^
Watershed area (km^2^)				0.46 (0.26)			
Elevation (m)							
Distance from sea (km)			–1.32 (0.79)		–0.64 (0.38)		
River width (m)		–1.0 (0.57)	3.60 (2.3)	–3.92 (2.1)		1.3 (0.71)	
Water depth (cm)							0.035 (0.015)
Water velocity (cm/sec)			–0.14 (0.15)	0.27 (0.19)			
Pebble size (cm)					0.22 (0.10)		–0.036 (0.020)
Slope angle (°)			0.70 (0.55)				
Canopy cover (%)			–0.34 (0.12)	–0.21 (0.11)			
AUC	1.00	0.88	1.00	1.00	1.00	0.92	

area under the curve (AUC) varies from 0 (totally mismatched model) to 1 (perfect model).

Levels of significance: ^***^P < 0.001; ^**^P < 0.01; ^*^P < 0.05.

Waterfall heights were determined using the laser range-finder. Waterfalls less than 3 m in height were disregarded because there were so many waterfalls less than 3 m and amphidromous fishes were always well distributed above them.

Multivariate analysis of variance (MANOVA) using the nine environmental parameters was used to determine whether integrated environmental conditions differed between the sites above and below/without waterfalls.

**Figure 2:**
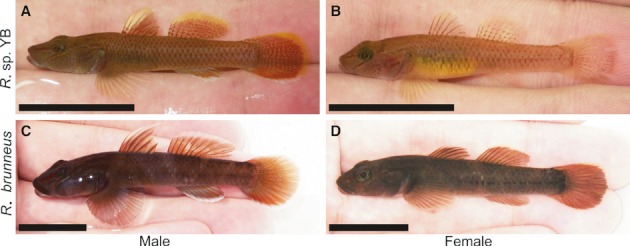
Photographic images of (A) male *Rhinogobius* sp. YB, (B) female *Rhinogobius* sp. YB, (C) male *Rhinogobius brunneus,* and (D) female *Rhinogobius brunneus*. Scale bar represents 20 mm.

A snorkeling survey (as in Watanabe and Ito 1999; Kano et al. 2010, 2011) was conducted at each study site to determine the fish fauna and the relative density of each fish species. The survey was also conducted between 5 and 15 June 2011. Most of the amphidromous fishes generally spawn from spring to summer after upstream migration from the sea (e.g., Maeda and Tachihara 2010) and a survey in June would be preferable to determine fish distribution. In the previous snorkeling survey studies, the detection rate ranged from 21.5 to 28.7% (standard error 3.3–8.8%) and 34.7% (95% confidence limit 27.0–43.1%) for *Pseudobagrus ichikawai* (demersal fish) (Watanabe and Ito 1999) and *Oncorhynchus masou ishikawae* (pelagic fish) (Kano et al. 2010), respectively. The survey, conducted only by Y. Kano to ensure methodological unity, involved swimming slowly upstream (50 m along the river; 50–150 m/h) with no overlapping or vacant searching areas. The surveyor tallied the observed fish of each species, using a waterproof notebook. The morphological difference of *R*. sp. YB and *Rhinogobius brunneus* was visually apparent (Fig. 2) (Akihito et al. 2002; Ohara et al. 2008) and the two can be easily distinguished on site.

### Habitat models

Habitat models of *R*. sp. YB, *R. brunneus*, *R*. sp. CB, *R*. sp. DL, Sicydiaphiinae spp., and *Kuhlia* spp. were constructed based on the data obtained from the evaluation of the river environment and the count data from the snorkeling survey. A generalized linear model (GLM) was used for the analysis with the presence/absence of the species/taxa as the dependent variable (1 or 0) and the ten environmental factors (Table 2) as explanatory variables. The presence/absence of a waterfall (Fig. 1; Table 1) below the site was treated as an explanatory dummy variable (1 or 0). Regression analyses were performed using the statistical software R (Ihaka and Gentleman 1996) with the “brglm” function (Kosmidis 2007) (family, binomial distribution with logit link function) because we could not perform standard analyses such as “glm” and “glmmML” due to the perfect separation. Logistic regression analysis was conducted for all possible sets of independent variables from a null model to a full model. Akaike's information criterion (AIC; Akaike 1974) was used for the model selection, and the model with the lowest AIC was defined as the best model.

A generalized linear mixed model (GLMM) was also used to analyze species richness using the “glmmML” function in R (family, Poisson distribution; random effect, river system). The best model was selected using the same method described above.

### Fin clipping and morphological evaluation

Fish were sampled for fin clipping and morphological evaluation between 12 and 22 April 2011. At selected sites where *R*. sp. YB and/or *R. brunneus* were distributed (colored sites in Fig. 1), adult males and females (four to six individuals for each sex for a total of 10 individuals at each site for each fish) were captured using a small hand net. To avoid biased sampling in genotype and morphology, individuals were caught randomly from different locations within the 50-m site. The sexes could be distinguished because males had larger mouth, longer snout, more colorful fins, and longer dorsal fin than females (Fig. 2) (Takahashi 2000; Takahashi and Kohda 2001). As for females, eggs could be clearly seen through their transparent bodies (Fig. 2B).

Each individual was anesthetized using clove oil diluted in water (50 ppm), and part of the left pectoral fin was cut and preserved in 100% ethanol for mtDNA analysis (described below). At several sites, fin samples of *Rhinogobius* sp. CB (cross-band type) (PN1, GT1, OM1, and YC1) and *Rhinogobius* sp. DL (depressed large-dark type) (UR4 and ML2) were obtained as references for the phylogenetic analysis. In total, 182 samples (*R*. sp. YB, 90; *R. brunneus*, 80; *R*. sp. CB, 6; *R*. sp. DL, 6) were obtained.

While the morphological/phenotypic difference of *R*. sp. YB and *R. brunneus* was apparent, a quantitative morphological evaluation was conducted to test the extent the two fishes were really separated in morphology, to what extent the *R*. sp. YB individuals displayed similarity to each other among the sites, and whether the phenotypes corresponded to genotypes. Nine morphological characters (Fig. S1) were evaluated from each of the sampled individuals of *R*. sp. YB and *R. brunneus* while under anesthesia. The nine characters were (*a*) darkened body, (*b*) black dots along the lateral line, (*c*) dense orange spots on the cheek, (*d*) thin extension of the first dorsal fin, (*e*) yellowness at the edge of the first dorsal fin, (*f*) yellowness at the edge of the second dorsal fin, (*g*) stripe pattern on the second dorsal fin, (*h*) yellowness at the upper backward edge of the tail fin, and (*i*) dark band on the base of the tail fin. A score of “0” or “1”was recorded depending on whether the character was absent or present, respectively, except in cases where a clear judgment was difficult and then a score of “0.5” was recorded.

### Morphological analyses

Score of each morphological character (Fig. S1; *a*–*i*) was compared between *R*. sp. YB and *R. brunneus* for each sex (because several characters were apparently different between the sexes) by the Mann–Whitney *U*-test. The level of significance was adjusted by the Bonferroni method (*P* = 0.05/18 = 0.0028).

Cluster analysis was conducted with the nine morphological characters for each sex. The Euclidean distances between all possible pairs of the individuals were then calculated using Ward's method with the “hclust” function in R.

### mtDNA sequencing and phylogenetic analysis

Fins obtained from the 182 individuals were used for the mtDNA analysis. Total DNA was isolated from the sample using DNA extraction kits (DNeasy Blood and Tissue kit; Qiagen). We targeted partial *ND5* and full cyt-*b* regions. Polymerase chain reaction (PCR) was conducted using the primer sets L12321-Leu (Miya and Nishida 2000) and H13396-ND5M (Miya and Nishida 2000) for the partial *ND5* region, and AJG15 and H5 (Akihito et al. 2000) for the full cyt-*b* region. The PCR products were sequenced using an automated DNA sequencer (3130xl Genetic Analyzer; Applied Biosystems). All sequences were deposited in DDBJ/GenBank (accession numbers: AB674598–AB674735). Data about the local populations were also deposited in GEDIMAP (Watanabe et al. 2010; Population IDs: P1358–P1378).

A statistical parsimony network of haplotypes was obtained using the program TCS ver. 1.21 (Clement et al. 2001), and it was then illustrated manually. Phylogenetic reconstructions were carried out using maximum-likelihood (ML) and neighbor-joining (NJ) models with the program MEGA5 (ver. 5.05) (Tamura et al. 2011). The “Tamura-Nei (Tamura and Nei 1993) + Γ (6 categories, Γ parameter = 0.14)” model was selected as the best model. Bootstrap analyses were performed with 1000 and 10,000 replicates for ML and NJ, respectively.

Hierarchical analysis of molecular variance (AMOVA; Excoffier et al. 1992) was performed using Arlequin ver. 3.5 (Excoffier and Lischer 2010) for *R*. sp. YB and *R. brunneus* separately to determine the genetic structures of populations. The variations were partitioned into two levels, that is, among sites and within respective sites. The Tamura-Nei model was used to determine the genetic distance. Statistical tests with φ-statistics were performed at 10,000 permutations.

Genetic distances (*D* with “Tamura-Nei + Γ”) among the separate *R*. sp. YB local populations (study sites were discriminated), the *R. brunneus* population (all individuals with no discrimination among study sites), the *R*. sp. CB population (all individuals), and the *R*. sp. DL population (all individuals) were determined using MEGA5.

### Waterfall height and genetic distance

We also tested whether waterfall height was correlated with the genetic distance of *R*. sp. YB local populations from *R. brunneus* because we assumed that waterfall height would reflect the isolation period as the waterfall was formed by ongoing geological land erosion (Larson et al. 2000). A simple liner regression model was applied based on the heights of waterfalls that isolated the *R*. sp. YB local populations from the *R. brunneus* population and the genetic distance between the two.

The coefficient (*C*) of the simple liner regression model obtained above and the evolutionary rate of *Rhinogobius* species (*R*) (3.8% per million years; Mukai et al. 2005) were used to estimate the erosion rate that formed the waterfalls. The erosion rate (*E*) was calculated as *E* = *R*/*C* and was applied to each waterfall of known height (Table 1). The mean genetic diversity of *R. brunneus* was also calculated using MEGA5 in order to know the genetic variation within *R. brunneus* as a reference for this analysis.

### Morphologic and genetic distance

We also tested whether morphological difference was correlated with genetic distance between each *R*. sp. YB local population and overall *R. brunneus* individuals. For each *R*. sp. YB individual, all possible Euclidian distances to *R. brunneus* individuals of the same sex were obtained and then averaged as the representative value of morphological distance from *R. brunneus*. The values were further averaged to yield a representative value for the site for each sex. A simple regression model was then applied between the genetic distance and the morphological distance for each sex. The level of significance was adjusted by the Bonferroni method (*P* = 0.05/2 = 0.025).

### Body size comparison in a sympatric habitat

At site YCR2 where *R*. sp. YB and *R. brunneus* coexisted (see Results), mature individuals were caught to compare the size between the two. We used a hand net to randomly catch 17 and 13 males and 20 and 12 females of *R*. sp. YB and *R. brunneus*, respectively. We measured the total length of each individual and compared the mean for each sex of *R*. sp. YB and *R. brunneus* by the Student's *t*-test. The level of significance was adjusted by the Bonferroni method (*P* = 0.05/2 = 0.025).

## Results

### Fish fauna and habitat models

Table S1 shows average values for environmental factors measured at each study site. There were no significant differences in integrated environment between sites above waterfalls and sites below waterfalls (MANOVA; Wilks Λ = 0.039, *P* > 0.05).

Over 15 species were observed during the snorkeling survey (Table 3). Several Sicydiaphiinae species were difficult to differentiate because juveniles were sometimes similar, and some species were unidentifiable. Other than *R*. sp. YB, the species observed were all amphidromous or brackish water fishes. *Rhinogobius* sp. YB was seen only at sites above the waterfalls, whereas most other fishes were usually found at the sites below or without waterfalls. Several amphidromous species were recorded above waterfalls (NK2, UR4, and YCR2), but species richness in such sites was low as compared with sites below or without waterfalls. YCR2 was the only site where *R*. sp. YB and *Rhinogobius brunneus* coexisted.

**Table 3:** Numbers of fish observed by the snorkeling survey at each study site (50 m along each stream) on Iriomote Island, Japan

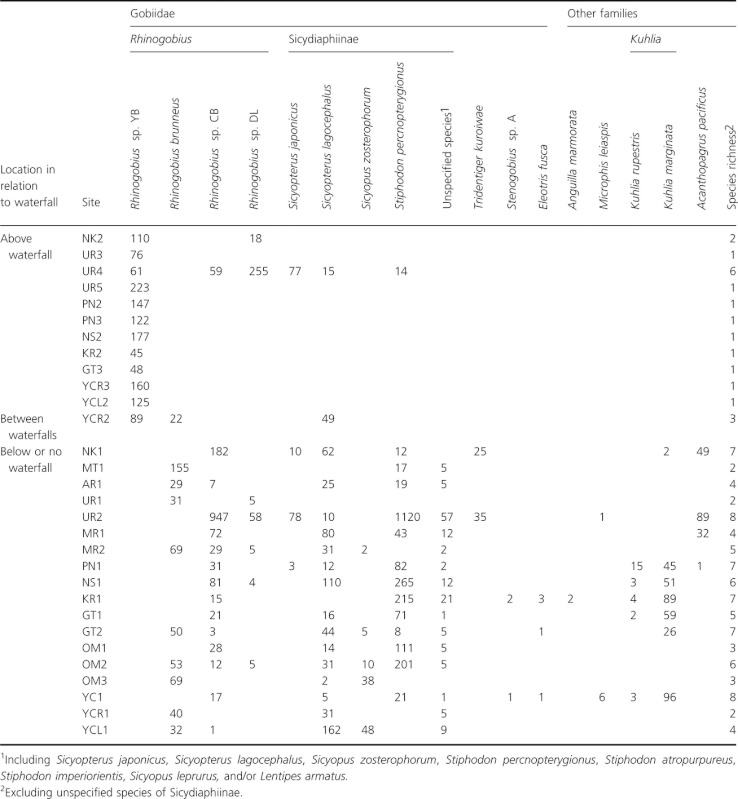

The GLM analysis and model selection by AIC showed that the presence of *R*. sp. YB was positively correlated with the presence of waterfalls below the sites but not with other factors (Table 2). In contrast, the presence of waterfalls below the sites was negatively correlated with the presence of *R. brunneus*, *Rhinogobius* sp. CB, Sicydiaphiinae spp., and *Kuhlia* spp., as well as with species richness (GLMM). Other factors were also selected in the best models, although none of these factors had a statistically significant effect.

### Morphological analyses

**Figure 3:**
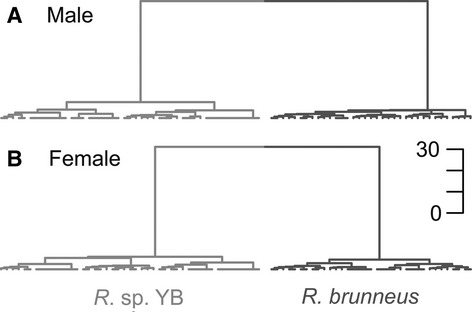
Results of cluster analysis for the nine morphological characters (Fig. S1 *a–i*) of *Rhinogobius* sp. YB (light gray) and *R. brunneus* (dark gray) for (A) males and (B) females of mature individuals. *Rhinogobius* sp. YB and *R. brunneus* formed distinct clusters without overlap.

With the exception of character *d* in females, all morphological characters showed a significant difference between *R*. sp. YB and *R. brunneus* in both sexes (*U*-test; all *P* < 0.0001; Table S2). In addition, cluster analysis clearly discriminated the two groups for each sex (Fig. 3). In both sexes, the individuals categorized as “*R*. sp. YB” in their appearance all belonged to *R*. sp. YB cluster, and those individuals noted as “*R. brunneus*” all belonged to *R. brunneus* cluster (Fig. 3).

### Phylogenetic analyses

The variable rates of *ND5* (0.0413; 39 of 945 bp) and cyt-*b* (0.0412; 47 of 1141 bp) sequences from *R*. sp. YB and *R*. *brunneus* were similar (Fisher's exact test, *P* > 0.05), so phylogenetic analyses were conducted using haplotypes that were a combination of sequences from the two regions (2086 bp in total).

**Figure 4:**
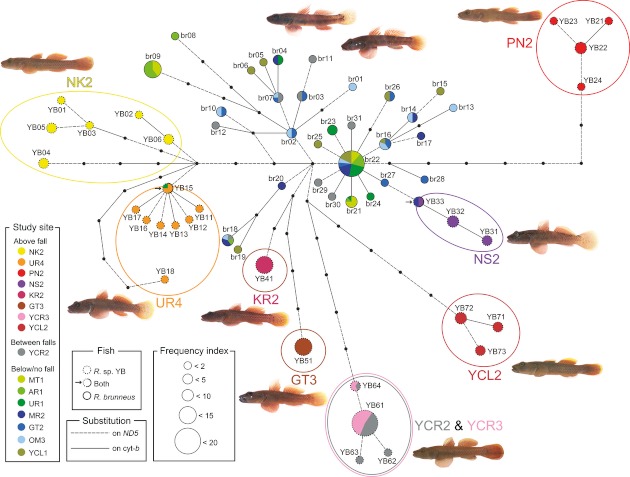
Statistical parsimony network of haplotypes for *Rhinogobius* sp. YB and *R. brunneus*. The colors correspond to the study sites on Figure 1. Dashed and solid circles indicate *R*. sp. YB and *R. brunneus*, respectively. Substitution of *ND5* and cyt-*b* are represented by dashed and solid lines, respectively. Haplotype names are shown next to each circle. Size of the circle indicates the frequency of individuals that belong to the haplotype. Photographic images of males of *R*. sp. YB (for each site) and *R. brunneus* (two individuals) are also shown. Note that all *R*. sp. YB local populations (shown by ellipses with their site color) are located on the edge of the network.

In total, 30, 33, 5, and 3 haplotypes were obtained from *R*. sp. YB, *R. brunneus*, *R*. sp. CB, and *Rhinogobius* sp. DL, respectively (Tables S3 and S4). Two haplotypes were shared by *R*. sp. YB and *R. brunneus*. The statistical parsimony network of haplotypes and the phylogenetic tree obtained are shown in Figs 4 and S2, respectively. With *R*. sp. YB, the haplotypes at respective sites formed separate clusters, whereas the haplotypes of *R. brunneus* were not site dependent (Figs. 4 and S2; Table S3). In addition, the haplotype clusters of the separate *R*. sp. YB local populations were all located on the edges of the network (Fig. 4), in contrast to a cluster of *R*. sp. YB in morphological evaluation (Fig. 3).

The AMOVA also confirmed these findings. The genetic variation in *R*. sp. YB was high among populations (90.3%; φ-statistics *P* < 0.0001) and low within populations (9.7%). In contrast, the genetic variation in *R*. *brunneus* was low among populations (6.4%; φ-statistics *P* < 0.01) and high within populations (93.6%).

Genetic distances among the groups are shown in Table S5. The distance between YCR2 and YCR3 for *R*. sp. YB was 0.0002, showing that *R*. sp. YB individuals from YCR3, and/or their descendants, had drifted downstream to YCR2 (see also Fig. 4). In contrast, the distance between PN2 and YCR2 had the highest value (0.0092) among the *R*. sp. YB local populations, and this was roughly one-half to one-third the distance between *R. brunneus* and *R*. sp. DL (0.0212), *R. brunneus* and *R*. sp. CB (0.0229), and *R*. sp. DL and *R*. sp. CB (0.0281).

### Waterfall height and genetic distance

**Figure 5:**
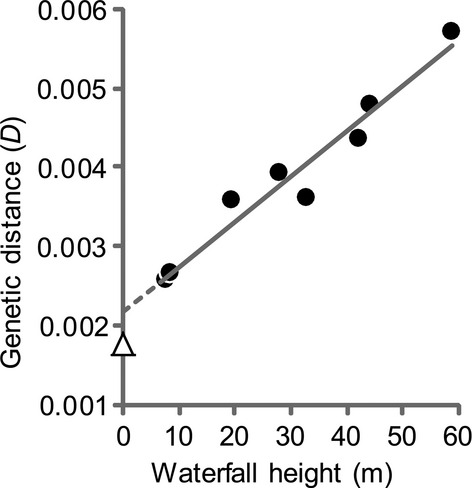
Relationship between waterfall height and genetic distance between each *Rhinogobius* sp. YB local population and *Rhinogobius brunneus* (simple liner regression model, slope = 0.000057, intercept = 0.0022, *F*_1,7_ = 90.1, *P* < 0.0001, *r*^2^ = 0.94). The unfilled triangle indicates the genetic variation within *R. brunneus* (0.00184), which roughly corresponds to the intercept. Data from YCR2 between waterfalls were omitted from this analysis because the *R*. sp. YB population at this site had probably drifted downstream from site YCR3 (see Results for details).

The distances between *R*. sp. YB local populations and the *R. brunneus* population varied from 0.0026 to 0.0057 (Table S5), and they were significantly positively correlated with waterfall height (Fig. 5; *P* < 0.0001). Data from YCR2 (between waterfalls) were omitted from this analysis because the *R*. sp. YB population of YCR2 had probably drifted from the YCR3 population (see above), making it unsuitable for the analysis. Using this information, the erosion rate forming the waterfalls was calculated to be 0.67 mm/year. Thus, the formation period of the waterfalls was estimated at ca. 11,000–88,000 years (Table 1).

### Morphologic and genetic distance

In both sexes, morphological Euclidian distance between two fishes was significantly positively correlated with genetic distance (Fig. S3) (Male, slope = 97.7, *F*_1,8_ = 12.8, *P* < 0.025, *r*^2^ = 0.65; female, slope = 141.9, *F*_1,8_ = 53.6, *P* < 0.001, *r*^2^ = 0.88).

### Size comparison in a sympatric habitat

The total length of adult male and female *R*. sp. YB individuals (male mean ± standard deviation [min–max]: 50.4 ± 5.0 [42–59] mm, female: 44.6 ± 4.9 [37–52] mm) was significantly smaller than that of *R. brunneus* (male: 79.2 ± 11.1 [63–94] mm, female: 59.3 ± 7.9 [47–75] mm) (Student's *t*-test; male: *t*_28_ = 9.5, female: *t*_30_ = 6.6, *P* < 0.0001 for both).

## Discussion

Based on the 10 environmental factors surveyed, waterfalls on Iriomote Island were the most important factor determining fish distribution. We had assumed that amphidromous fishes would be generally distributed below waterfalls because the waterfalls are a physical barrier. However, the absence of *Rhinogobius* sp. YB below waterfalls is notable because downstream migration via waterfalls should be possible and physical conditions of the habitat do not appear to affect distribution (Table 2). It is possible that *R*. sp. YB is vulnerable to interspecific competition and those individuals that drift down fail to survive (except at YCR2 as discussed below). Selection pressure from interspecific competition is low above the waterfalls because of the relative absence of other species (Table 3) (e.g., Magurran 2005; Crispo et al. 2006; Covich et al. 2009; Blob et al. 2010), although the waterfalls did not exclude all amphidromous individuals, as seen at sites NK2, UR4, and YCR2, which were situated above relatively low waterfalls. Another landlocked goby, *Rhinogobius* sp. TO (Tokai area type), is distributed among ponds in central Japan and is reported to be threatened with extinction by alien species, such as the largemouth bass *Micropterus salmoides* (Suzuki and Mukai 2010). Extinction of native species due to interspecific competition from newly introduced species has been attributed to native species not having evolved a competitive ability (e.g., Cox 1999; Mooney and Hobbs 2000).We conclude, therefore, that the waterfalls of Iriomote Island provide anomalous habitats for *R*. sp. YB by preventing large numbers of migrating fishes; waterfalls are one of the main factors determining distribution and diversity of fish on the island.

The site YCR2 was the only site where *R*. sp. YB and *Rhinogobius brunneus* coexisted. This may have been due to the ongoing downstream dispersal of *R*. sp. YB from YCR3 and the impediment to migration of several amphidromous fishes that were potential competitors at Mayarock Falls as this site only permits the upstream migration of *R. brunneus* and *Sicyopterus lagocephalus* (Table 3). This exceptional sympatric distribution prompted us to examine whether *R*. sp. YB (at least at YCR2) and *R*. *brunneus* are different species, and the results of mtDNA analysis clearly separated two different groups with a genetic distance of 0.0048 (Fig. 4; Table S5). In the stickleback, the reproductive isolation between landlocked (small) and anadromous (large) populations is ecologically achieved by body size difference (McKinnon et al. 2004). Similarly, landlocked *R*. sp. YB was significantly smaller than amphidromous *R. brunneus* in both sexes. We suspect reproductive isolation between *R*. sp. YB and *R. brunneus* might result from body size difference as well as other morphological characters (e.g., Genner et al. 2007).

Phylogenetic analysis confirmed the independent evolution of *R*. sp. YB in its respective habitats, suggesting that the population has evolved repeatedly above waterfalls. In contrast, amphidromous *R. brunneus* had a relatively high genetic diversity at each site and a low genetic divergence among sites, suggesting that *R. brunneus* from the various sites is a single population. This is in keeping with its amphidromous life history that allows genetic interaction beyond the rivers (e.g., Cook et al. 2009). These results support the suggestion that *R*. sp. YB has evolved repeatedly from *R. brunneus* (Nishida 1994; Ohara et al. 2008) deriving from its genetic variation (unfilled triangle in Fig. 5). However, haplotypes were somewhat mixed between NK2 and UR4 in a single clade (Fig. 4; Fig. S2), suggesting that genetic introgression and/or incomplete genetic sorting may have occurred at some stage in history. This perhaps could have been from topographic changes in the watershed between the Nakara River (the third longest river in the island) and the Urauchi River (the longest river in the island) (Fig. 1). In addition, several subclades (e.g., YB04 and YB18 in Fig. 4) were found in these clades. We suspect that several waterfalls exist along the tributaries of the Nakara River (above NK2) and Urauchi River (above UR4) and that several *R*. sp. YB local populations have independently evolved at these locations. Some members of these populations would have moved downstream, resulting in various subclades.

Our results indicate that genes developed independently but the separate *R*. sp. YB local populations retained similar morphology, suggesting a typical case of parallel evolution. The evolutionary diversification of respective *R*. sp. YB local populations from *R. brunneus* was achieved rapidly after the formation of the waterfalls (ca. 11,000–88,000 years). The transporter hypothesis based on standing genetic variation and parallel evolution predicts that speciation is potentially more rapid than that expected from new mutations (Hermisson and Pennings 2005; Barrett and Schluter 2008; Schluter and Conte 2009). Our results, therefore, might be showing such rapid parallel speciation. Parallel evolution by landlocking has been mainly studied in the sticklebacks (e.g., Hagen and MacPhail 1970; Rundle et al. 2000; Taylor and McPhail 2000; Reusch et al. 2001; McKinnon et al. 2004), and our study is the first detail report of parallel evolution from the Gobiidae, one of the largest families of fish. It is also notable that the spatial scale of our study is considerably smaller than that in the stickleback studies. Putative parallel evolution is occurring, or has occurred, above waterfalls on a small island; diversification is evident even in tributaries with the same drainage (YCR3 and YCL2 in the Yuchin River).

Cluster analysis showed that respective *R*. sp. YB local populations were generally similar morphologically; however, finer scale analysis indicated slight but significant morphological differentiation among the local populations. Local populations of *R*. sp. YB having high genetic distance from *R. brunneus* (i.e., above high waterfalls) also had high morphological distance from *R. brunneus*. The two fishes were genetically separated at YCR2, but the haplotypes of *R*. sp. YB at UR4 (YB15) and NS2 (YB33), sites that were separated by relatively low waterfalls, were also shared by *R. brunneus* (Fig. 4; Table S3). It is likely that *R*. sp. YB individuals drifted or moved downstream to UR4 and NS2 and mated with *R. brunneus* individuals because the genetic features and morphology of two fishes are still relatively similar when compared with further evolved local populations, such as those at PN2 and YCR3. We cannot confirm whether *R*. sp. YB and *R. brunneus* are different species, but our results indicate that speciation/evolution of multiple phase is simultaneously occurring at different locations across the island because of its heterogeneous geography, that is, variations in waterfall height.

One of the representative morphological characters of *R*. sp. YB is the yellow color of the fins; we cannot provide a hypothesis as to why the fins became yellow at present. In guppies, however, males are colorful at low predation sites, whereas they are less colorful at high predation sites (Endler and Houde 1995). A similar phenomenon might have occurred in *R*. sp. YB. In either case, we suspect that the morphological differentiation of the two fishes might be attributed to presence/absence of interspecific interactions, also leading to the morphological similarity of *R*. sp. YB beyond the genetic divergence of this fish.

Cliffs are often formed from heterogeneous geologic strata when a harder rock type overlies a weaker rock that is then weathered (Larson et al. 2000). The same mechanism is applicable to the formation of waterfalls. When a river flows over resistant bedrock, erosion occurs slowly, whereas downstream erosion occurs more rapidly. The difference in erosion rates leads to the formation of a geologic step (Carrick 1982; Fuller 2012). In the current study, the erosion rate of waterfalls on Iriomote Island was calculated as 0.67 mm/year. The land erosion rate in the Japanese Islands is hypothesized to be correlated with the standard deviation of elevation, regardless of the lithologic character (Fujiwara et al. 1999); the erosion rate is high (2–3 mm/year) in rugged areas but low (0.07–0.12 mm/year) in flat areas. On Iriomote Island, the standard deviation for elevation is 113.9 m (see Methods), so the erosion rate was roughly estimated at 0.3–1.0 mm/year on the basis of a relationship plot in Fujiwara et al. (1999). Thus, our estimated waterfall erosion rate of 0.67 mm/year does not conflict with this estimation, but further surveys and more precise data are necessary to provide a more precise timeline. Nevertheless, we can at least conclude that the evolution of *R*. sp. YB is correlated with the process of waterfall formation on the island: each local population of *R. brunneus* was once isolated and landlocked upstream by waterfall formations, consequently evolving to *R*. sp. YB in each site.

Waterfalls locally decrease the diversity of fish fauna/genes above the waterfalls (e.g., Martin-Smith and Laird 1998; Carlsson and Nilsson 2001; Castric et al. 2001; Costello et al. 2003; Mazzoni et al. 2006; Guy et al. 2008), but on a wider landscape scale, habitat diversity is increased by waterfalls, generating various gene types above respective waterfalls. Waterfalls have also influenced the survival of *R*. sp. YB on the island and possibly have affected the survival of aquatic organisms other than fish by limiting predator populations, as reported in freshwater shrimps (Covich et al. 2009). We conclude that waterfalls have important ecological/evolutionary roles in river ecosystems and can even lead to speciation. Additional case studies will elucidate the relationship between waterfalls and fluvial organisms.

A question that remains to be answered is whether the separate *R*. sp. YB local populations above the waterfalls are the same species. We expect to follow up this study with one comparing the detailed ecology, behavior, and morphology among the *R*. sp. YB local populations, as well as conducting breeding experiments in the laboratory. If the local populations are the same species, then genetic differences do not necessarily define a species, at least in terms of mtDNA. If the populations were different species, it follows that indigenous species are being generated or have been generated above separate waterfalls on this small island.

## Supporting Information

**Figure S1.** Photographic images of *Rhinogobius* sp. YB and *R. brunneus*.

**Figure S2.** Phylogenetic tree of *Rhinogobius* sp. YB, *R. brunneus*, *R*. sp. CB, and *R*. sp. DL constructed by the maximum-likelihood method.

**Figure S3.** Relationship between genetic distance and morphological Euclidian distance from *Rhinogobius brunneus* for each *R*. sp. YB local population.

**Table S1.** Average values for environmental factors measured at each study site (50 m along each stream) on Iriomote Island, Japan.

**Table S2.** Score frequency of morphological characters (*a*–*i*) for each sex of *Rhinogobius* sp. YB and *R. brunneus*.

**Table S3.** Haplotype frequency of *Rhinogobius* sp. YB and *R. brunneus* at each study site.

**Table S4.** Haplotype frequency of *Rhinogobius* sp. CB and *R*. sp. DL at each study site.

**Table S5.** Genetic distances among separate *Rhinogobius* sp. YB local populations, *R*. brunneus, *R*. sp. DL, and *R*. sp. CB.
