# Supplementary material for: Waterfalls drive parallel evolution in a freshwater goby
Source: Ecol Evol. 2012 Jul 1;2(8):1805–17. doi: 10.1002/ece3.295 (PMC3433985; doi:10.1002/ece3.295)
Supplement: Supplementary file 1 [file ece30002-1805-SD1.pdf]

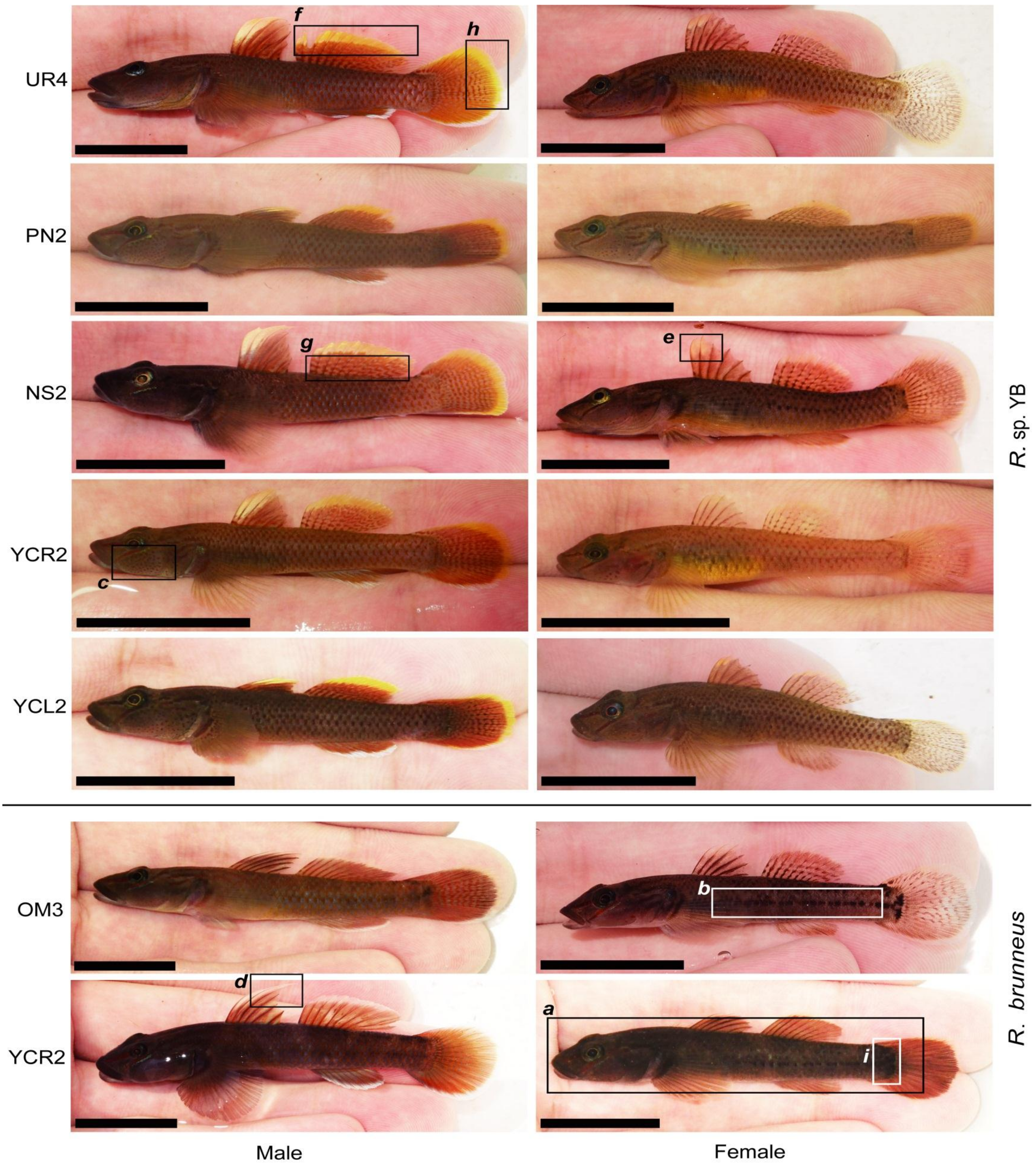

Figure S1. Photographic images of *Rhinogobius* sp. YB (above) and *Rhinogobius brunneus* (below). Typical characters of *R.* sp. YB and *R. brunneus* are shown in squares with letters. Scale bar represents 20 mm.

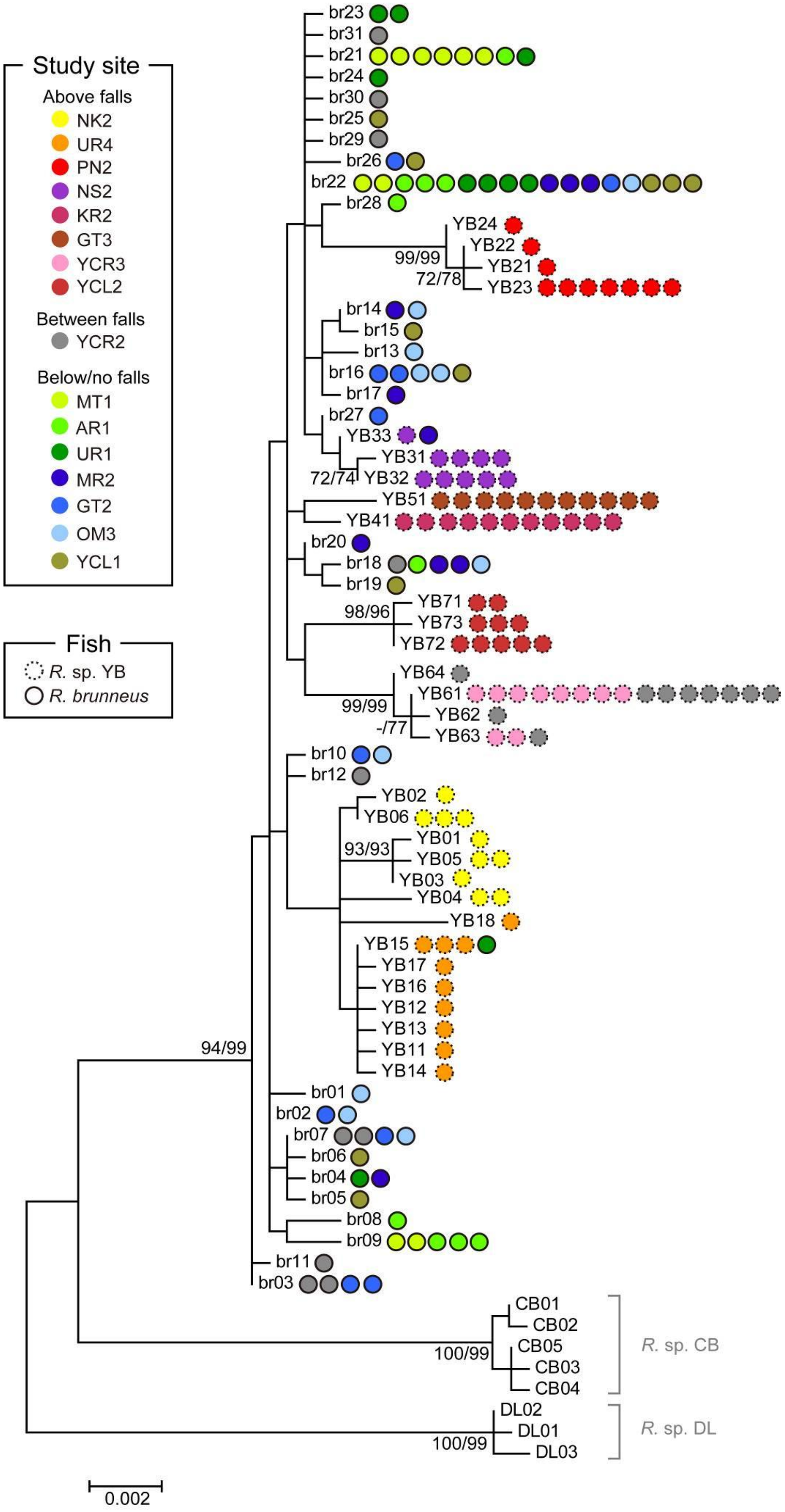

Figure S2. Phylogenetic tree of *Rhinogobius* sp. YB, *R.* brunneus, *R.* sp. CB, and *R.* sp. DL constructed by the ML method. The numbers along each branch show bootstrap percentages for ML (1000 permutations) and NJ method (10,000 permutations). Bootstrap values over 70% are shown. The distance bar shows 0.002. The haplotype names correspond to those in Tables S2 and S3. Each circle represents an individual and its site (colour) and species (*R.* sp. YB, dashed circle; *R.* brunneus, solid circle).

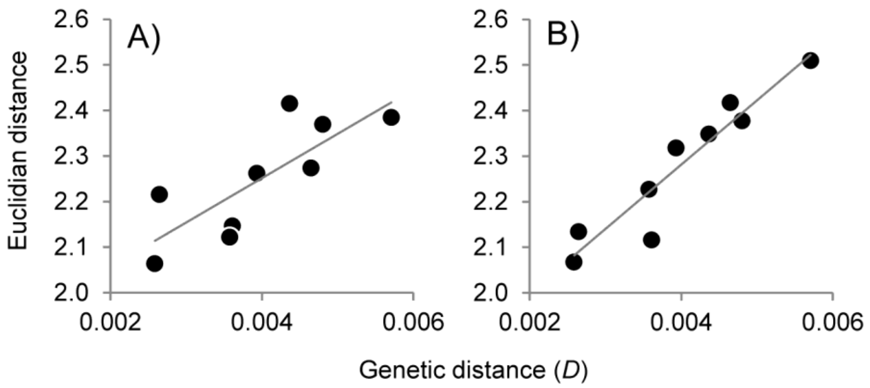

Figure S3. Relationship between genetic distance and morphological Euclidian distance from *Rhinogobius brunneus* for each *R.* sp. YB local population. A) Male, slope = 97.7, $F_{1,8}$ = 12.8, $P < 0.025$, $r^2 = 0.65$; B) female, slope = 141.9, $F_{1,8}$ = 53.6, $P < 0.001$, $r^2 = 0.88$.

Table S1. Environmental factors measured at each study site (50 m along each stream).

Location in relation to waterfall	Site	GIS			River conditions in the field					
		Watershed area (km^2)	Elevation (m)	Distance from sea (km)	River width (m)[1]	Water depth (cm)[1]	Water velocity (cm/sec)[1]	Pebble size (cm)[1]	Slope angle (°)	Canopy cover (%)[1]
Above waterfall	NK2	6.2	36	5.8	3.6	60.0	38.6	36.7	2.0	50.0
	UR3	0.4	65	9.9	1.4	32.8	15.5	28.9	10.6	83.5
	UR4	37.8	49	10.1	7.4	102.0	36.2	65.3	1.5	34.5
	UR5	1.2	96	12.5	2.4	25.0	17.9	17.3	4.0	73.0
	PN2	2.8	134	1.4	2.7	37.6	18.6	23.9	3.7	83.0
	PN3	2.2	155	2.1	2.4	27.3	17.0	20.8	1.6	80.0
	NS2	3.6	36	1.8	4.0	33.2	15.9	23.8	4.0	49.9
	KR2	0.9	21	0.7	2.4	27.8	14.6	25.2	1.7	89.6
	GT3	1.1	48	0.8	1.1	21.3	18.6	23.0	14.0	95.6
	YCR3	0.1	331	2.8	2.3	24.5	10.2	17.2	1.7	85.6
	YCL2	0.7	259	2.7	2.9	26.9	10.3	24.0	1.9	88.9
	Mean	5.2	111.8	4.6	2.9	38.0	19.4	27.8	4.2	74.0
Between waterfalls	YCR2	1.7	73	1.5	3.5	27.0	28.3	32.5	4.4	80.4
Below or no waterfall	NK1	6.3	12	5.7	4.7	67.7	34.1	50.1	2.0	55.7
	MT1	0.6	20	1.7	2.8	19.2	14.9	14.9	5.1	93.3
	AR1	1.7	27	0.5	1.3	32.8	15.5	28.9	10.6	86.1
	UR1	0.3	33	8.5	1.0	17.9	10.8	20.4	14.6	68.1
	UR2	41.8	22	9.1	9.9	117.6	45.0	95.6	0.8	40.1
	MR1	3.0	5	1.0	3.2	25.4	30.2	30.3	6.8	86.3
	MR2	2.4	63	2.1	2.1	34.1	27.2	33.7	8.9	81.5
	PN1	2.9	52	1.2	3.8	40.4	20.9	36.5	6.7	81.5
	NS1	3.6	27	1.7	2.3	36.3	20.5	34.0	5.8	77.6
	KR1	0.9	10	0.7	2.2	27.2	12.0	16.9	3.1	82.0
	GT1	1.3	9	0.4	3.2	26.9	11.8	16.9	4.5	82.8
	GT2	1.2	33	0.7	2.1	24.5	18.8	19.6	3.9	74.1
	OM1	2.1	16	0.3	2.8	32.1	21.6	29.7	0.5	77.1
	OM2	2.0	36	0.8	2.5	24.9	18.6	26.4	1.2	55.6
	OM3	0.9	92	1.5	1.6	32.3	17.2	35.5	5.6	88.8
	YC1	2.1	14	0.3	3.9	26.4	20.1	20.5	2.6	81.7
	YCR1	1.8	60	1.4	2.4	29.0	29.2	28.9	5.7	83.3
	YCL1	0.9	195	2.4	2.5	33.8	13.4	31.9	9.8	85.8
	Mean	4.2	40.4	2.2	3.0	36.0	21.2	31.7	5.5	76.7

[1]Average value.

Table S2. Score frequency of morphological characters (*a*–*i*) for each sex of *Rhinogobius* sp. YB and *R. brunneus*.

Character	Male							Female						
	R. sp. YB			*R. brunneus*			*U*-test	*R.* sp. YB			*R. brunneus*			*U*-test
	"0"	"0.5"	"1"	"0"	"0.5"	"1"		"0"	"0.5"	"1"	"0"	"0.5"	"1"	
a	41	4	0	2	13	25	*	33	12	0	1	20	19	*
b	45	0	0	9	25	6	*	34	11	0	0	3	37	*
c	0	4	41	20	18	2	*	15	19	11	39	1	0	*
d	11	26	8	1	13	26	*	45	0	0	40	0	0	NS
e	4	22	19	38	1	1	*	0	1	44	20	19	1	*
f	0	6	39	39	1	0	*	0	2	43	38	2	0	*
g	0	3	42	30	8	2	*	0	7	38	19	16	5	*
h	0	0	45	34	6	0	*	0	2	43	36	4	0	*
i	43	2	0	18	14	8	*	33	12	0	0	5	35	*

A score of "0" or "1"was absent or present of the character, respectively, except in cases of difficult judgement and then a score of "0.5" was given. Levels of significance: *$P < 0.0001$.

Table S3. Haplotype frequency of *Rhinogobius* sp. YB (underlined) and *R. brunneus* at each study site.

Haplotype	Accession number (*ND5*, cyt-*b*)	Site																Total
		Above waterfall								Between falls	Beneath or no waterfall							
		NK2	UR4	PN2	NS2	KR2	GT3	YCR3	YCL2	YCR2	MT1	AR1	UR1	MR2	GT2	OM3	YCL1	
YB01	AB674598, AB674667	1																1
YB02	AB674599, AB674668	1																1
YB03	AB674600, AB674669	1																1
YB04	AB674601, AB674670	2																2
YB05	AB674602, AB674671	2																2
YB06	AB674603, AB674672	3																3
YB11	AB674604, AB674673		1															1
YB12	AB674605, AB674674		1															1
YB13	AB674606, AB674675		1															1
YB14	AB674607, AB674676		1															1
YB15	AB674608, AB674677		3										1					4
YB16	AB674609, AB674678		1															1
YB17	AB674610, AB674679		1															1
YB18	AB674611, AB674680		1															1
YB21	AB674612, AB674681			1														1
YB22	AB674613, AB674682			7														7
YB23	AB674614, AB674683			1														1
YB24	AB674615, AB674684			1														1
YB31	AB674616, AB674685				4													4
YB32	AB674617, AB674686				5													5
YB33	AB674618, AB674687				1									1				2
YB41	AB674619, AB674688					10												10
YB51	AB674620, AB674689						10											10
YB61	AB674621, AB674690							8		7								15
YB62	AB674622, AB674691									1								1
YB63	AB674623, AB674692									1								1
YB64	AB674624, AB674693							2		1								3
YB71	AB674625, AB674694								2									2
YB72	AB674626, AB674695								5									5
YB73	AB674627, AB674696								3									3
br01	AB674628, AB674697															1		1
br02	AB674629, AB674698														1	1		2
br03	AB674630, AB674699									2					2			4
br04	AB674631, AB674700												1	1				2
br05	AB674632, AB674701																1	1
br06	AB674633, AB674702																1	1
br07	AB674634, AB674703									2					1	1		4
br08	AB674635, AB674704											1						1
br09	AB674636, AB674705										2	3						5
br10	AB674637, AB674706														1	1		2
br11	AB674638, AB674707									1								1
br12	AB674639, AB674708									1								1
br13	AB674640, AB674709															1		1
br14	AB674641, AB674710													1		1		2
br15	AB674642, AB674711																1	1
br16	AB674643, AB674712														2	2	1	5
br17	AB674644, AB674713													1				1
br18	AB674645, AB674714									1		1		2		1		5
br19	AB674646, AB674715																1	1
br20	AB674647, AB674716													1				1
br21	AB674648, AB674717										6	1	1					8
br22	AB674649, AB674718										2	3	4	3	1	1	3	17
br23	AB674650, AB674719												2					2
br24	AB674651, AB674720												1					1
br25	AB674652, AB674721																1	1
br26	AB674653, AB674722														1		1	2
br27	AB674654, AB674723														1			1
br28	AB674655, AB674724											1						1
br29	AB674656, AB674725									1								1
br30	AB674657, AB674726									1								1
br31	AB674658, AB674727									1								1
N																		
R. sp. YB		10	10	10	10	10	10	10	10	10								90
R. brunneus										10	10	10	10	10	10	10	10	80
GEDIMAP ID																		
R. sp. YB		P1358	P1359	P1360	P1361	P1362	P1363	P1364	P1365	P1366								
R. brunneus										P1367	P1368	P1369	P1370	P1371	P1372	P1373	P1374	

Arrays of the haplotypes were deposited in DDBJ/EMBL-Bank/GenBank, whereas haplotype frequency and additional information for respective populations were deposited in GEDIMAP (Watanabe et al. 2010).

Table S4. Haplotype frequency of *Rhinogobius* sp. CB and *R.* sp. DL at each site.

Species	Haplotype	Accession number (*ND5*, cyt-*b*)	Site					
			UR4	MR2	PN1	GT2	OM1	YC1
R. sp. CB	CB01	AB674659, AB674728				1		
	CB02	AB674660, AB674729					1	
	CB03	AB674661, AB674730						1
	CB04	AB674662, AB674731			1			
	CB05	AB674663, AB674732			1	1		
R. sp. DL	DL01	AB674664, AB674733		1				
	DL02	AB674665, AB674734	2	2				
	DL03	AB674666, AB674735	1					
GEDIMAP ID								
R. sp. CB					P1375	P1376	P1377	P1378
R. sp. DL			P1379	P1380				

Arrays of the haplotypes were deposited in DDBJ/EMBL-Bank/GenBank with their accession numbers, whereas haplotype frequency and additional information for respective populations were deposited in GEDIMAP (Watanabe et al. 2010).

Table S5. Genetic distances among separate *Rhinogobius* sp. YB local populations, *R. brunneus*, *R.* sp. DL, and *R.* sp. CB.

	Species	Site	1	2	3	4	5	6	7	8	9	10	11	12
1	*R*. sp. YB	NR2		0.0024	0.0077	0.0041	0.0044	0.0054	0.0061	0.0060	0.0062	0.0039	0.0228	0.0254
2		UR4	0.0024		0.0076	0.0038	0.0040	0.0051	0.0062	0.0060	0.0057	0.0036	0.0221	0.0247
3		PN2	0.0077	0.0076		0.0063	0.0068	0.0078	0.0092	0.0091	0.0086	0.0057	0.0280	0.0277
4		NS2	0.0041	0.0038	0.0063		0.0037	0.0046	0.0059	0.0057	0.0054	0.0026	0.0225	0.0249
5		KR2	0.0044	0.0040	0.0068	0.0037		0.0029	0.0053	0.0051	0.0045	0.0026	0.0224	0.0226
6		GT3	0.0054	0.0051	0.0078	0.0046	0.0029		0.0062	0.0060	0.0058	0.0036	0.0235	0.0250
7		YCR2	0.0061	0.0062	0.0092	0.0059	0.0053	0.0062		0.0002	0.0060	0.0048	0.0251	0.0266
8		YCR3	0.0060	0.0060	0.0091	0.0057	0.0051	0.0060	0.0002		0.0058	0.0046	0.0249	0.0264
9		YCL2	0.0062	0.0057	0.0086	0.0054	0.0045	0.0058	0.0060	0.0058		0.0044	0.0228	0.0257
10	*R. brunneus*		0.0039	0.0036	0.0057	0.0026	0.0026	0.0036	0.0048	0.0046	0.0044		0.0212	0.0229
11	*R*. sp. DL		0.0228	0.0221	0.0280	0.0225	0.0224	0.0235	0.0251	0.0249	0.0228	0.0212		0.0281
12	*R*. sp. CB		0.0254	0.0247	0.0277	0.0249	0.0226	0.0250	0.0266	0.0264	0.0257	0.0229	0.0281	

Amphidromous *R. brunneus*, *R.* sp. YB, *R.* sp. DL and *R.* sp. CB were each treated as a single population regardless of the sampling sites, whereas landlocked *R.* sp. YB was treated at the local population level.
