# Supplementary figures and images for: Waterfalls drive parallel evolution in a freshwater goby
Source: Ecol Evol. 2012 Jul 1;2(8):1805–17. doi: 10.1002/ece3.295 (PMC3433985; doi:10.1002/ece3.295)

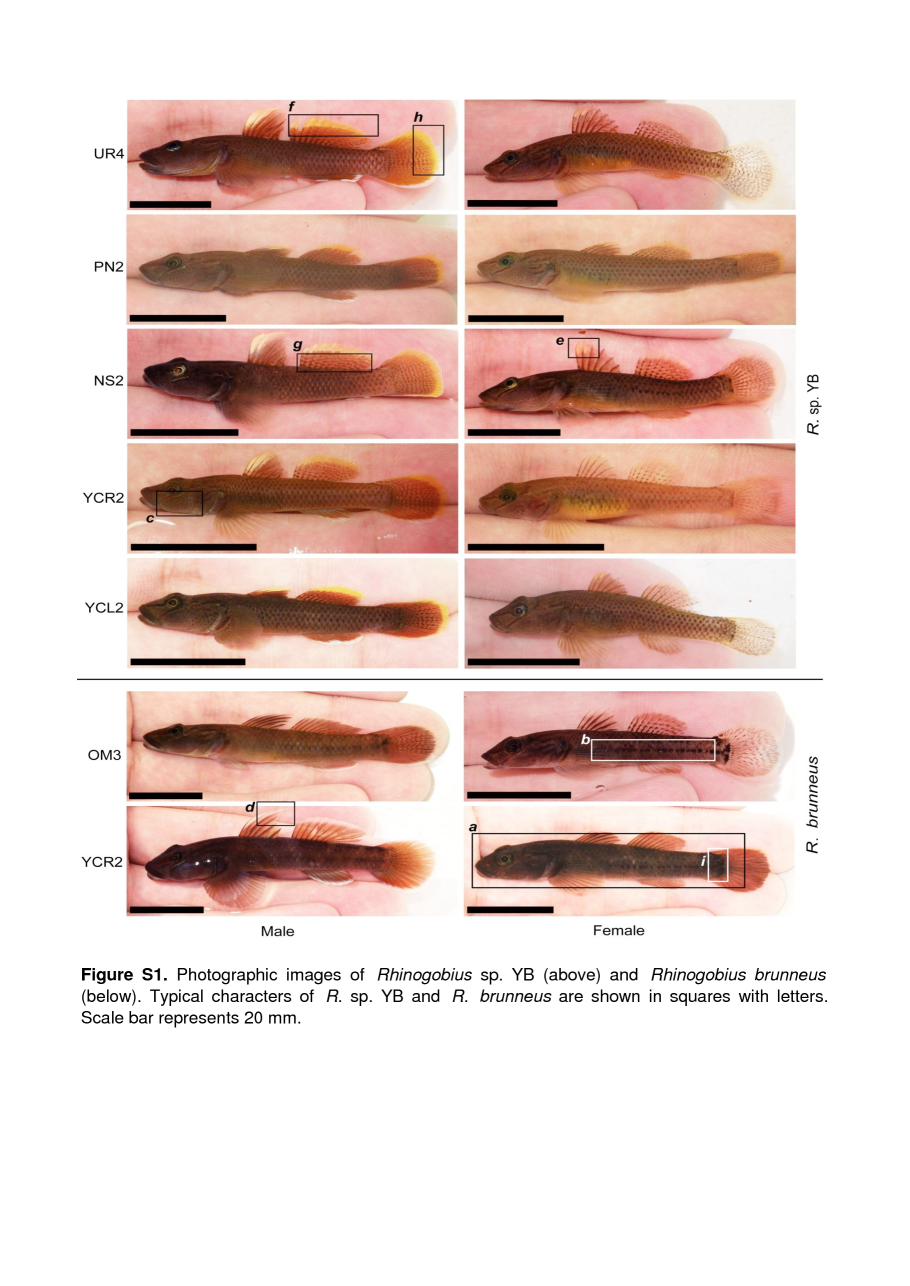

Supplement: Supplementary file 2 [file ece30002-1805-SD2.png]
